# Increased extracellular volume, reduced stress perfusion, and worse systolic function in Wilson’s disease

**DOI:** 10.1016/j.jocmr.2025.102669

**Published:** 2025-12-12

**Authors:** Rebecka Steffen Johansson, Csenge Fogarasi, Peter Kellman, Andreas Kindmark, Jannike Nickander

**Affiliations:** a Department of Clinical Physiology, Karolinska University Hospital, and Department of Molecular Medicine and Surgery, Karolinska Institutet, Stockholm, Sweden; b National Heart, Lung, and Blood Institute, National Institutes of Health, Bethesda, Maryland, USA; c Department of Medical Sciences, Endocrinology and Mineral Metabolism, Uppsala University, and Uppsala University Hospital, Uppsala, Sweden

**Keywords:** Wilson’s disease, Coronary microvascular dysfunction, CMR multiparametric mapping, CMR perfusion mapping, Stress perfusion

## Abstract

**Background:**

Wilson’s disease (WD) causes intracellular copper accumulation due to a genetic defect in the copper-transporting protein ATP7B. Cardiac involvement has been reported even in young WD patients; however, pathophysiological mechanisms remain unclear. This study aimed to comprehensively assess the myocardium in WD patients without cardiac symptoms using multiparametric cardiovascular magnetic resonance imaging (CMR), including quantitative stress perfusion mapping and strain analysis.

**Methods:**

WD patients and healthy volunteers underwent multiparametric 1.5T CMR, including cine, native T1, native T2, extracellular volume (ECV), adenosine stress perfusion mapping, and late gadolinium enhancement (LGE) imaging. Left and right ventricle (LV, RV) mass and volumes, global native T1, native T2, ECV, rest and stress perfusion, myocardial perfusion reserve (MPR), strain measures and liver native T1 were compared. LGE images were assessed visually. Disease type and duration, medications, and cardiovascular risk factors were recorded. Symptoms of myocardial ischemia were quantified with Seattle Angina Questionnaire-7.

**Results:**

WD patients (n = 17, 34 [29–55] years, 8/17 (47%) female) and healthy volunteers (n = 17, 33 [29–52] years, 8/17 (47%) female, *p* = ns for both) were included. There were no differences in cardiovascular risk factors or medications. LV ejection fraction was lower in WD patients (57 [55–61] vs 62 [57–67] %, *p* = 0.02), and LV global circumferential strain was mildly worse (−18 [−19 to (−17)] vs −20 [−21 to (−18)] %, *p* = 0.005), otherwise there were no differences in LV or RV mass or function. WD patients had lower stress perfusion and MPR (2.95 [2.74–3.29] vs 3.81 [2.67–4.45] mL/min/g, and 3.3 [3.1–3.8] vs 5.0 [2.9–5.4]), while ECV was higher (29 [28–30] vs 26 [26–29] %), *p*<0.05 for all, but there were no other differences in multiparametric mapping results. ECV did not correlate with strain parameters. ECV was associated with WD and sex but not age (WD β = 2.58%, male sex β = −0.03%, model R^2^ 0.41, *p*<0.05 for all). LGE was present in the RV insertion point in 12/17 (71%) of WD patients.

**Conclusions:**

In this study, stable WD patients without apparent cardiac symptoms have early signs of diffuse fibrosis, coronary microvascular dysfunction, and worse systolic function. However, this study is limited by small sample size limiting further subgroup analysis, lack of both longitudinal clinical data and biopsies, not allowing for correlation of CMR findings to histopathology.

## Graphical abstract

**Figure:**
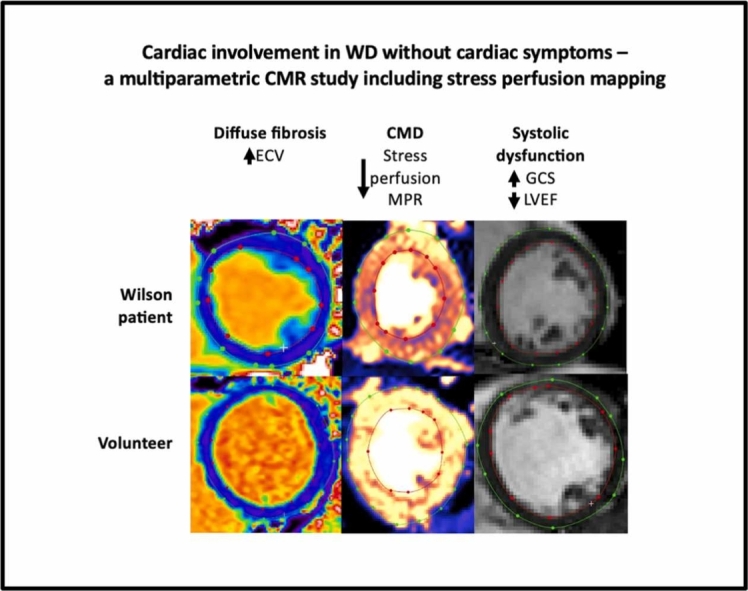
The main findings of our study comparing WD patients without cardiac symptoms with healthy volunteers using multiparametric CMR, including stress perfusion, are higher global ECV, reduced stress perfusion and MPR as well as mildly impaired GCS and lower LVEF in WD patients. *CMD* coronary microvascular dysfunction, *CMR* cardiovascular magnetic resonance imaging, *ECV* extracellular volume, *GCS* global circumferential strain, *LVEF* left ventricular ejection fraction, *MPR* myocardial perfusion reserve, *WD* Wilson’s disease

## 1. Introduction

Wilson’s disease (WD) causes intracellular copper accumulation, due to a genetic defect in the copper-transporting protein ATP7B [1]. Cardiac involvement has been reported even in early WD, with electrocardiographic (ECG) abnormalities, rhythm disturbances, hypertrophy, and heart failure [2]. However, pathophysiological mechanisms are unclear. This study aimed to comprehensively assess the myocardium in WD patients without cardiac symptoms using multiparametric cardiovascular magnetic resonance imaging (CMR), including perfusion mapping and strain analysis.

## 2. Methods

WD patients (Leipzig score ≥4), one year symptomatically and biochemically stable, were prospectively recruited at the national WD center at Uppsala University Hospital, Sweden. Exclusion criteria were standard contraindications to adenosine and contrast-enhanced CMR. Previously examined sex- and age-matched healthy volunteers were included. Medical history, medications, ECG, hematocrit, and creatinine, were obtained. WD type and severity were assessed by an experienced endocrinologist. Cardiac symptoms were quantified with Seattle Angina Questionnaire-7 (SAQ-7). The Swedish Ethical Review Authority granted ethical approval for the study. All participants provided written informed consent.

The detailed study protocol is available at MedRxiv [3]. In short, CMR was performed from January 2022 to March 2023 at Karolinska University Hospital, Stockholm, Sweden, using a MAGNETOM Aera 1.5T scanner (Siemens Healthineers, Erlangen, Germany). Cine images in three long-axis (LA) and short-axis (SA) slices were acquired. Quantitative perfusion maps were obtained by first-pass perfusion imaging in three SA slices during adenosine stress and rest, following abstinence from caffeine and nicotine for 24 and 12 h. Native T1 maps and post-contrast T1 maps were acquired in three SA slices, rendering extracellular volume (ECV) maps following calibration by hematocrit. Native T2 maps were acquired in three SA slices. Post-contrast late gadolinium enhancement (LGE) images were obtained in three LA and SA slices.

Anonymized images were analyzed offline using Segment (vers 3.3 Medviso AB, Lund, Sweden). The myocardium was delineated in each cine stack in end-systole and end-diastole, to obtain left and right ventricle (LV, RV) mass and volumes. Global longitudinal, circumferential, and radial strain (LVGLS, RVGLS, LVGCS, LVGRS) were acquired using the feature-tracking strain module. Left and right atrioventricular plane displacement (AVPD) were obtained using the AVPD-module. The myocardium was delineated in SA native T1, native T2, ECV, and perfusion maps, applying a 10% erosion margin, segmental values were exported and averaged to global values. Global liver T1 was obtained by averaging three midventricular regions of interest. LGE images were evaluated visually and scarring determined as % of LV mass (LVM).

Data were presented as median [interquartile range] and numbers (percentages), and compared using the independent t-test, Mann–Whitney U test, or Fisher’s exact test, following evaluation of normality using the Shapiro-Wilk test. Mass and volumes were indexed to body surface area (Mosteller formula). Segments with LGE were excluded from ECV analysis. Rate pressure product (RPP) was calculated as the product of heart rate and systolic blood pressure, myocardial perfusion reserve (MPR) as the ratio of stress to rest perfusion. Relationships between ECV and strain were explored using Pearson or Spearman correlation. The effect of sex, age, WD disease, and its duration, severity (mild/severe), type (hepatic/neurological) on ECV, stress perfusion, and MPR were explored using multivariate regression.

Inter- and intra-observer agreement were analyzed for native T1, T2, ECV, rest and stress perfusion, MPR, LV ejection fraction (LVEF), LVGCS, LVGRS, and LVGLS, by two independent observers in all 17 WD patients and by one observer in 10 WD patients. Two-way random effects with absolute agreement was applied. The intra-class correlation coefficient ranged 0.82–1.00 (*p*<0.05 for all). Our previously published data on healthy volunteers showed 16 participants needed to detect 0.78 mL/min/g difference in stress perfusion (power 80%, alpha 0.05). Data were gathered in Excel (Microsoft, Redmond, Washington, USA) and analysis performed in SPSS Statistics (version 28, IBM Corp., Armonk, New York). The significance level was *p*<0.05.

## 3. Results

Clinical characteristics and CMR findings of WD patients (n = 17, 34 [29−55] years, 8/17 (47%) female) and healthy volunteers (n = 17, 33 [29−52] years, 8/17 (47%) female, *p* = ns for both) are shown in Table 1. No one had hypertension, hyperlipidemia, smoked, or used angiotensin-converting enzyme inhibitors, angiotensin receptor blockers, or statins. WD was classified as mild (n = 12) or severe (n = 5), hepatic (n = 12), neurological (n = 4), or without main type (n = 1). Disease duration at CMR was 23 [15−34] years. Treatments included trientine (n = 9), zinc (n = 4), or penicillamine (n = 1), liver transplant (n = 1), or no treatment due to mild disease (n = 2) (Table 1, Appendix). One WD patient had sinus tachycardia (101 beats per minute), one had a small intraventricular conduction defect, one had frequent supraventricular and ventricular extra beats, otherwise ECGs were normal (ECG missing for 3 patients). SAQ-7 summary score for WD patients was 100 [95−100] (data missing for 2).

**Table 1:** Clinical characteristics and CMR findings

	WD patients (n = 17)	Volunteers (n = 17)	*p*
Female sex, n (%)	8 (47%)	8 (47%)	1.00
Age (years)	34 [29–55]	33 [29–52]	0.80
Height (cm)	173 [171−183]	175 [165−186]	0.79
Weight (kg)	80 [61–90]	70 [65–72]	0.13
BSA(m^2^)	2.0 [1.7−2.1]	1.9 [1.7−1.9]	0.19†
Creatinine (mmol/l)	72 [69–94]	71 [57–84]	0.12†
Hematocrit (%)	40 [38–42]	43 [41–46]	**0.02**
Angina pectoris, n (%)	1 (6%)	0 (0%)	0.49
Diabetes mellitus, n (%)	1 (6%)	0 (0%)	0.49
Smoking previously, n (%)	5 (31%)	7 (41%)	0.72
Beta blockers, n (%)	1 (6%)	0 (0%)	0.49
CCBs, n (%)	1 (6%)	0 (0%)	0.49
LVMi (g/m^2^)	46 [42–57]	53 [50–58]	0.15
LVEDVi (mL/m^2^)	93 [85−112]	97 [82−100]	0.66
LVESVi (mL/m^2^)	38 [33–54]	37 [32–43]	0.29†
LVSVi (mL/m^2^)	54 [50–58]	57 [53–64]	0.39
LVEF (%)	57 [55–61]	62 [57–67]	**0.02**
CO (l/min)	6.8 [5.8-8.2]	6.6 [5.5-8.8]	0.93†
LVGLS (%)	-19 [−19 to (−18)]	-19 [−20 to (−17)]	0.22†
LVGCS (%)	-18 [−19 to (−17)]	-20 [−21 to (−18)]	**0.005**†
LVGRS (%)	40 [36–44]	44 [37–49]	0.23
Left AVPD (mm)	-14 [−16 to (−13)]	-15 [−15 to (−14)]	0.34
Native T1 myocardium (ms)	1003 [959−1031]	1008 [968−1023]	0.67
Native T1 liver (ms)	561 [518−601]	555 [531−570]	0.46†
Native T2 (ms)	49 [47–51]	49 [48–50]	0.49
ECV (%)	29 [28–30]	26 [26–29]	**0.003**
RPP stress	11140 [10117−12469]	10974 [9750−11484]	0.64
Perfusion stress (mL/min/g)	2.95 [2.74−3.29]*	3.81 [2.67−4.45]	**0.017**
Perfusion rest (mL/min/g)	0.86 [0.77−1.00]*	0.85 [0.79−0.98]	0.89†
MPR	3.3 [3.1−3.8]*	5.0 [2.9−5.4]	**0.044**

Clinical characteristics and CMR findings are presented as n (%) or median [interquartile range], *p*-values denote Fisher’s exact test or the independent t-test, p-values marked † denote the Mann–Whitney U test, * perfusion maps are missing in one WD patient due to contrast failure. *AVPD* atrioventricular plane displacement, *BSA* body surface area, *CCBs* calcium channel blockers, *CMR* cardiovascular magnetic resonance imaging, *CO* cardiac output, *ECV* extracellular volume, *LVEDV* left ventricular end-diastolic volume, *LVEF* left ventricular ejection fraction, *LVESV* left ventricular end-systolic volume, *LVSV* left ventricular stroke volume, *LVGLS* left ventricular global longitudinal strain, *LVGCS* left ventricular global circumferential strain, *LVGRS* left ventricular radial strain, *LVM* left ventricular mass, *MPR* myocardial perfusion reserve, *RPP* rate pressure product, *WD* Wilson’s disease. Volumes and mass marked i are indexed to BSA according to the Mosteller formula.

**Fig. 1:**
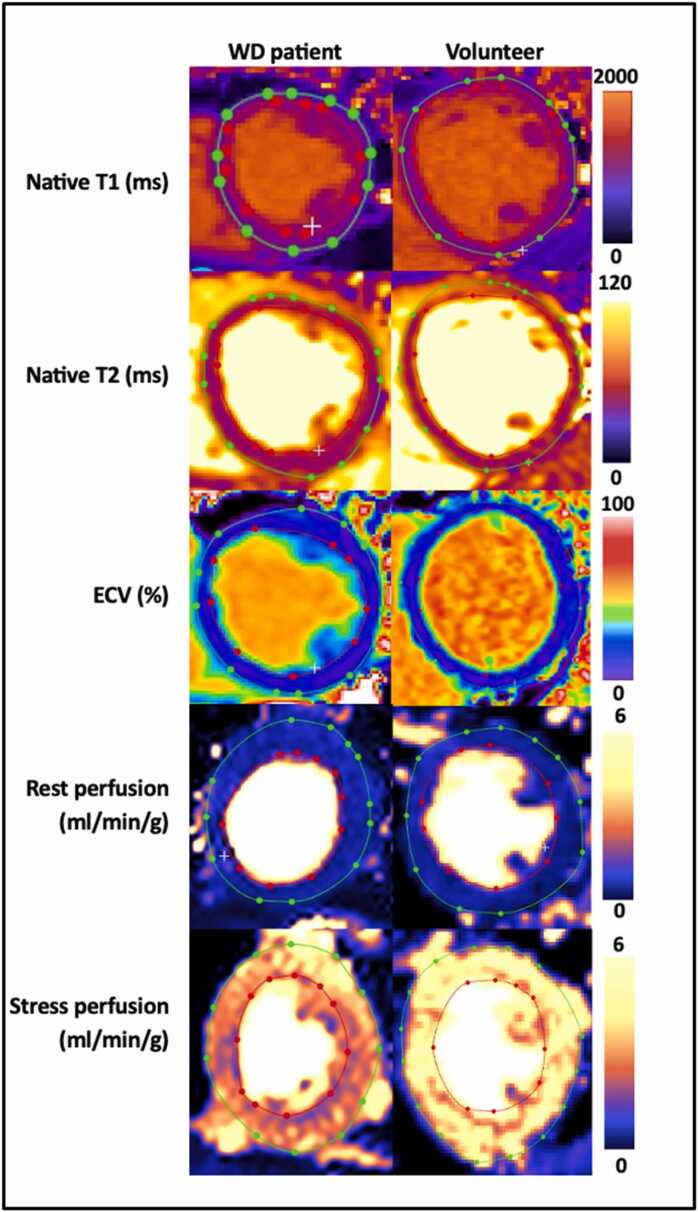
Multiparametric CMR in a WD patient and volunteer. Midventricular short-axis native T1 (ms), native T2 (ms), ECV (%), rest and stress perfusion maps (mL/min/g), including segmentation delineating endocardial (red) and epicardial (green) borders. *ECV* extracellular volume, *WD* Wilson’s disease, *CMR* cardiovascular magnetic resonance.

**Fig. 2:**
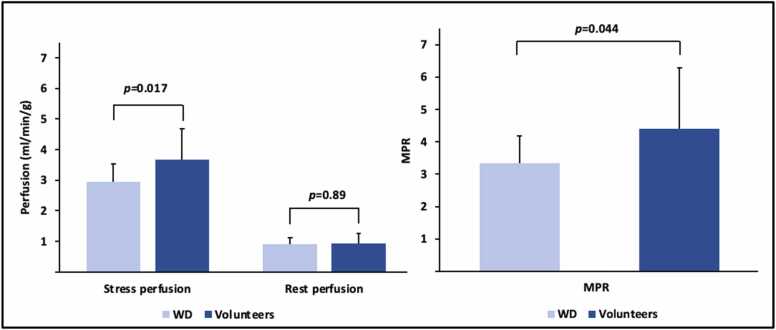
Perfusion and MPR in WD patients and volunteers. Bar charts show mean and error bars show standard deviation of stress and rest perfusion (mL/min/g) and MPR, together with p-values denoting the independent t-test. *MPR* myocardial perfusion reserve, *WD* Wilson’s disease.

WD patients had lower LVEF (57 [55−61] vs 62 [57−67] %, *p* = 0.02) and LVGCS was mildly worse (−18 [−19 to (−17)] vs −20 [−21 to (−18)] %, *p* = 0.005), there were no other differences in biventricular volumes or LVM (Table 2, Appendix). A WD patient and a healthy volunteer are shown in Fig. 1. Perfusion maps are missing in one WD patient due to contrast failure. WD patients had lower stress perfusion and MPR (2.95 [2.74–3.29] vs 3.81 [2.67–4.45] mL/min/g, *p* = 0.017, and 3.3 [3.1–3.8] vs 5.0 [2.9–5.4], *p* = 0.044), Fig. 2. ECV was higher (29 [28−30] vs 26 [26−29] %, *p* = 0.003). ECV did not correlate with GLS, GCS, or GRS (data not shown). ECV was associated with WD and sex (WD β = 2.58%, male sex β = −0.03%, model R^2^ 0.41, *p*<0.05 for all). Moreover, stress perfusion was associated with WD, sex, and age (WD β = −0.65, male sex β = −0.009, age β = −0.023 mL/min/g, model R^2^ 0.40, *p*<0.05 for all).

RV insertion point LGE (RVIP-LGE) was present in 12/17 (71%) WD patients. Basal inferolateral non-ischemic midmural LGE (1% of LVM) with native T2 52 ms was found in one WD patient; one had basal inferolateral non-ischemic midmural and epicardial LGE (3%) with native T2 59 ms; one had basal lateral non-ischemic midmural LGE (5%) with native T2 57 ms in the scar. Two had no LGE.

## 4. Discussion

This study investigated early cardiac involvement in WD using comprehensive CMR, including stress perfusion mapping and strain analysis, and showed signs of coronary microvascular dysfunction (CMD), diffuse fibrosis, and worse systolic function (Graphical Abstract).

Previous CMR studies in WD show inconsistent results, likely due to differences in sex, age, ethnicity, sample size, disease stage, and treatment. Signal intensity changes on brain magnetic resonance imaging in WD likely reflect edema or necrosis rather than intracellular copper. Elevated native T1 and T2 reflect active inflammation, edema, or necrosis; elevated ECV indicates diffuse fibrosis or infiltration. Previous studies have shown increased native T1, native T2, and ECV with normal LVM, biventricular volumes, and strain [1], without LGE or with RVIP-LGE [1], [4]. We showed normal global native T1 and T2 with increased ECV, RVIP-LGE, and non-ischemic LGE with elevated native T2 in the scar, possibly indicating active focal inflammation despite long-term clinical stability and normal liver native T1. Elevated native T2 with myocarditis-pattern and LGE has been shown in acute WD exacerbation, with normalization of native T2 following decoppering [5]. Native T1 and ECV are higher in neurological WD, which is considered more advanced, and heart failure may be present [4]. Most patients in our cohort had mild, stable WD, which may explain normal native T1 and T2.

We showed focal and diffuse fibrosis but normal LVM. Previous studies report both signs of fibrosis with normal LVM [1] or LGE with increased LVM [5], [6]. LVEF and LVGCS were mildly affected, while previous studies showed normal strain [1], impaired LVGRS [6], or LVGCS [4]. RV dysfunction has been reported [5], [6], which was not present. Autopsy studies have shown cardiac hypertrophy, interstitial and replacement fibrosis, intramyocardial small vessel sclerosis, and inflammation in newly diagnosed or chronic WD, some with sudden death [7]. In hypertrophic cardiomyopathy similarly characterized by hypertrophy, interstitial and replacement fibrosis, and dysplastic intramyocardial arterioles, decoppering improves T1, ECV, LVM, and LVGLS by reducing diffuse fibrosis [8]. We demonstrated CMD by impaired stress perfusion and MPR, which in the absence of obstructive coronary disease may be caused by oxidative stress, inflammation, fibrosis, small vessel sclerosis, or dysplasia with capillary rarefaction and hypertrophy [9]. Although not studied in the heart, copper accumulation causes oxidative stress, inflammation, apoptosis, and fibrosis [10], which may explain diffuse fibrosis, CMD, and LV dysfunction.

Limitations include small sample size limiting further subgroup analysis, limited longitudinal clinical data, lack of biopsies not allowing correlation of CMR findings to histopathology, and potential referral bias. However, our findings may warrant cardiac evaluation at WD diagnosis and follow-up to avoid future cardiac complications, particularly as sudden cardiac death may occur even in early WD [2]. Future studies should include larger populations in multicenter longitudinal follow-up of both imaging and clinical parameters.

## 5. Conclusions

In this study, stable WD patients without apparent cardiac symptoms have early signs of diffuse fibrosis, CMD and worse systolic function. However, this study is limited by small sample size limiting further subgroup analysis, lack of longitudinal clinical data and biopsies, not allowing for correlation of CMR findings to histopathology.

## Funding

The study was funded in part by The Swedish Society of Medicine, 10.13039/100010823Tore Nilsson Foundation, 10.13039/501100006285Magnus Bergvalls Stiftelse, and Swedish Heart and Lung Foundation. R.S.J. is funded by The Swedish Society of Medicine, Swedish Heart and Lung Foundation and the Region of Stockholm, Sweden. C.F. is funded by The Swedish Society of Medicine, and Swedish Heart and Lung Foundation. A.K. is funded by the Region of Uppsala. J.N. is funded by Swedish Heart and Lung Foundation and the Region of Stockholm.

## Author contributions

**Rebecka Steffen Johansson:** Writing – review & editing, Writing – original draft, Visualization, Software, Methodology, Investigation, Formal analysis. **Csenge Fogarasi:** Writing – review & editing, Validation, Investigation. **Peter Kellman:** Visualization, Supervision, Software, Conceptualization. **Andreas Kindmark:** Resources, Investigation, Conceptualization. **Jannike Nickander:** Writing – review & editing, Supervision, Resources, Funding acquisition, Data curation, Conceptualization.

## Ethics approval and consent

The Swedish Ethical Review Authority has granted ethical approval for the study (DNR 2019–06287, with amendments 2021–05508-02, 2022–06995-02 and for volunteers 2015/2106–31/1 and 2014/131–31/1, 2017/2415–32/1, 2021–06837-02). All participants provided written informed consent.

## Consent for publication

All participants provided written informed consent to publication of individual data on group level and to publish anonymized images. Consent forms are documented in the clinical notes of the participants and original forms are held in our research office and are available for the Editor-in-Chief for review.

## Declaration of competing interests

The authors declare the following financial interests/personal relationships which may be considered as potential competing interests: Previously, J.N. has received minor speaker compensation from Sanofi-Genzyme for work unrelated to the current study. A.K. has research grants funded by Sanofi-Genzyme, Takeda, Orphalan, BioMarin and has received consultant or speaker compensation from Amgen, UCB, Sanofi-Genzyme, Takeda, Orphalan, BioMarin, Chiesi, Abacus, and Amicus. R.S.J., C.F. and P.K. declare no relationships with industry. The Karolinska University Hospital has a research and development agreement with Siemens Healthineers.

## Figures and Tables

**Table 1:** Clinical characteristics of WD patients.

	All (n = 17)	Male (n = 9)	Female (n = 8)
Disease duration at CMR (years)	23 [15–34]	15 [7–33]	30 [22–40]
Disease severity, n			
Mild	12	5	7
Severe	5	4	1
Main WD manifestation, n			
None	1	0	1
Neurological	4	2	2
Liver	12	7	5
Treatment, n			
None	2	0	2
Zinc	4	1	3
Penicillamine	1	1	0
Trientine	9	6	3
Liver transplant	1	1	0

Clinical characteristics of WD patients presented as median [interquartile range], and frequencies, for all and grouped by sex. *CMR* cardiovascular magnetic resonance, *WD* Wilson’s disease.

**Table 2:** RV volumes and function.

CMR findings	WD patients (n = 17)	Volunteers (n = 17)	*p*
RVEDVi (mL/m^2^)	89 [72–105]	93 [79–99]	0.93
RVESVi (mL/m^2^)	34 [22–41]	30 [24–36]	0.63
RVSVi (mL/m^2^)	55 [50–60]	57 [52–65]	0.55
RVEF (%)	62 [58–66]	66 [58–70]	0.40
RVGLS (%)	-18 [−22 to (−17)]	-20 [−23 to (−18)]	0.16
Right AVPD (mm)	-20 [−22 to (−17)]	-21 [−23 to (−17)]	0.63†

CMR findings are presented as median [interquartile range], *p*-values denote the independent t-test, *p*-values marked † denote the Mann–Whitney U test. *AVPD* atrioventricular plane displacement, *CMR* cardiovascular magnetic resonance imaging, *RVEDV* right ventricular end-diastolic volume, *RVEF* right ventricular ejection fraction, *RVGLS* right ventricular global longitudinal strain, *RVESV* right ventricular end-systolic volume, *RVSV* right ventricular stroke volume, *WD* Wilson’s disease.

## Data Availability

Upon reasonable request, data supporting the findings in this study are available from the corresponding author.
